# Arguments for a neuroorthopaedic strategy in upper limb arthrogryposis

**DOI:** 10.1186/1749-7221-8-9

**Published:** 2013-10-17

**Authors:** Jörg Bahm

**Affiliations:** 1 Euregio Reconstructive Microsurgery Unit, Franziskus hospital, Morillenhang 27, D 52074, Aachen, Germany

**Keywords:** Arthrogryposis, Upper limb, Nerve transfer, Brachial plexus

## Abstract

We present two children with a diagnosis of upper limb arthrogryposis and report on findings about brachial plexus exploration and a nerve transfer procedure to reanimate elbow flexion. Although the etiology of arthrogryposis multiplex congenita remains unknown and multifactorial, it can be worthful to explore the brachial plexus in the affected upper limb and to perform selective motor nerve transfers on morphologically well developed but not sufficiently innervated target muscles, like the biceps brachialis, brachialis, deltoid and supra-/infraspinatus muscles. This strategy may reduce the necessity of later muscle transfers and improves the overall functional status of the affected limb(s).

## Introduction

Arthrogryposis multiplex congenita (AMC) is a well known clinical entity of unknown, certainly multifactorial etiology [1,2]. Akinetic, neuro- and/or myopathic forms are described and associations with syndromes like the whistling face syndrome (Freeman- Sheldon) are particular clinical entities.

AMC is characterized by a variable functional impairment of upper and/or lower limbs due to muscular hypotrophy and imbalance and joint ankylosis since birth. The treatment is orthopaedic and surgical according to the severity. In upper limb impairment, some authors claim an early surgical correction by joint releases and muscle transfers, to reanimate essential motor functions like the elbow flexion [2]. Little is known about the underlying pathophysiology and eventual peripheral or central nerve damages [1]. There is generally no spasticity in the affected limbs.

We present two clinical observations allowing insights in nerve variations and a possible strategy for an early functional improvement. Further studies and observations should strengthen the hypothesis of treatable proximal motor nerve alterations in some cases of upper limb AMC.

## Case presentations

Girl patient 1 was born as the first child of a mother with a known uterus malformation (septum) by caesarian section. Immediately after birth, hypotrophy of the partially paralyzed left upper limb was observed. Due to the lack of active shoulder and elbow movements in a medially rotated arm, the diagnosis of severe upper obstetric brachial plexus palsy was hypothesized and the child was presented at our consultation.

**Figure 1:**
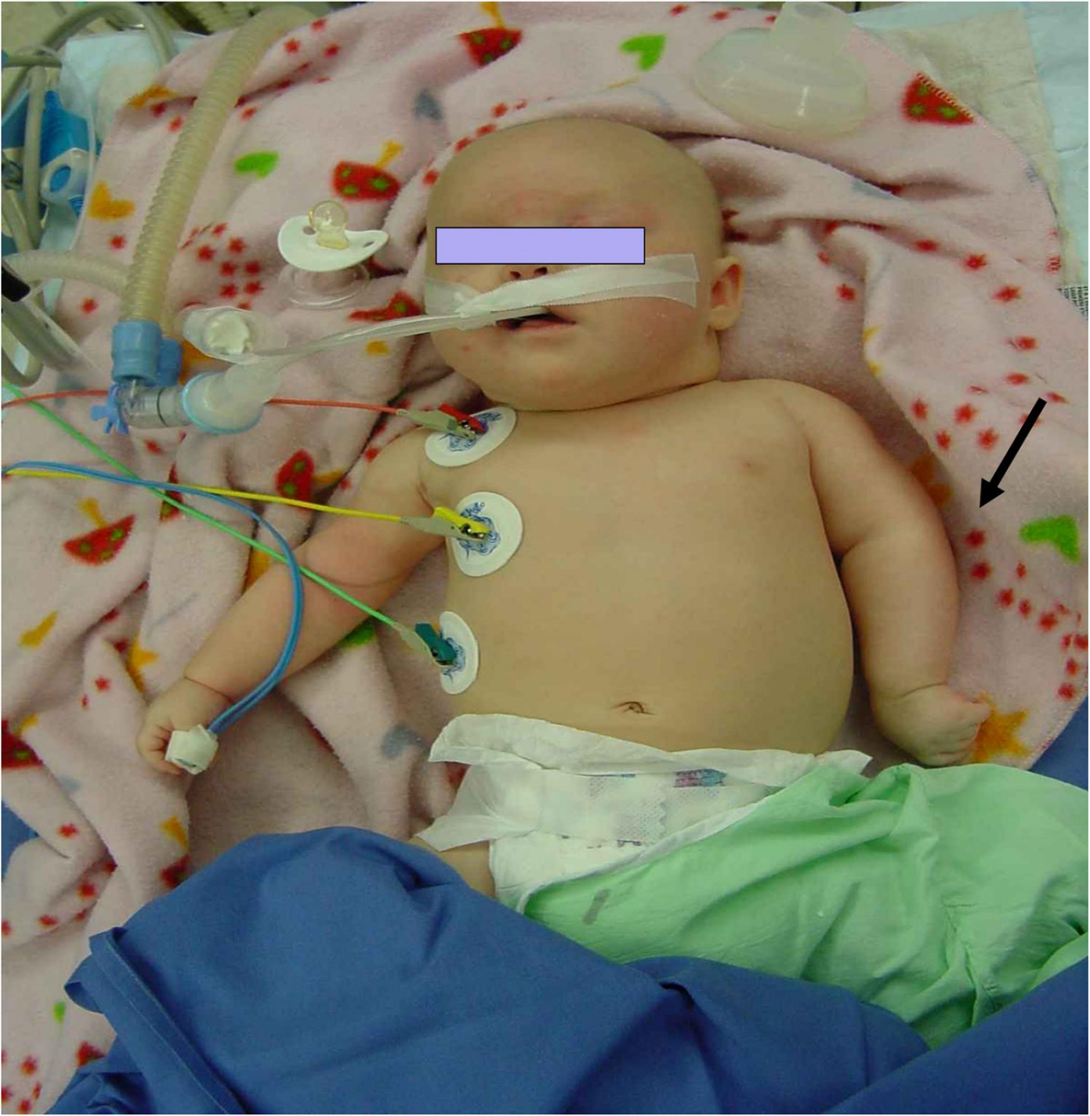
**Patient 1 preoperatively.** Left upper limb palsy, medial rotation position and hypotrophy.

As the palsy was severe and did not show any clinical improvement at three months, a surgical exploration of the left brachial plexus was performed when she was aged four months (Figure 1) under the hypothesis of a possible intrauterine malposition of the child (a very rare condition discussed in obstetric palsy).

**Figure 2:**
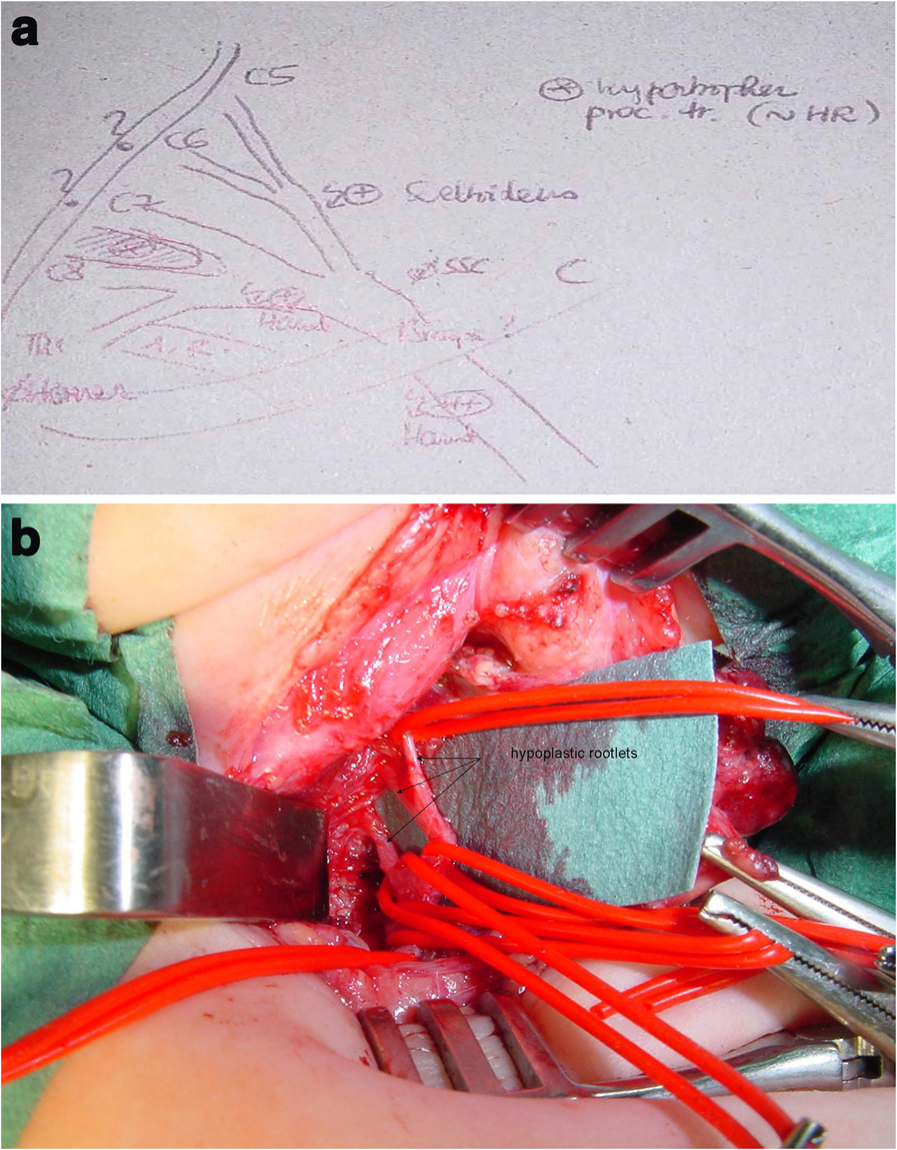
**Patient 1 intraoperative view of the brachial plexus.** Root and trunk hypoplasia

The brachial plexus showed to be hypoplastic, with thin roots and trunks (Figure 2a and b). There were no anterior – posterior divisions at the trunk level; the lower and middle trunk could not be individualized and the suprascapular nerve was absent. On intraoperative direct electrical stimulation, the upper trunk gave some answers in the deltoid muscle, but no biceps activity could be identified. The lower trunk stimulation showed some finger flexion activity. No further reconstruction was performed at this age and the child was followed for over eight years, confirming finally a typical unilateral upper limb development consistent with AMC.

**Figure 3:**
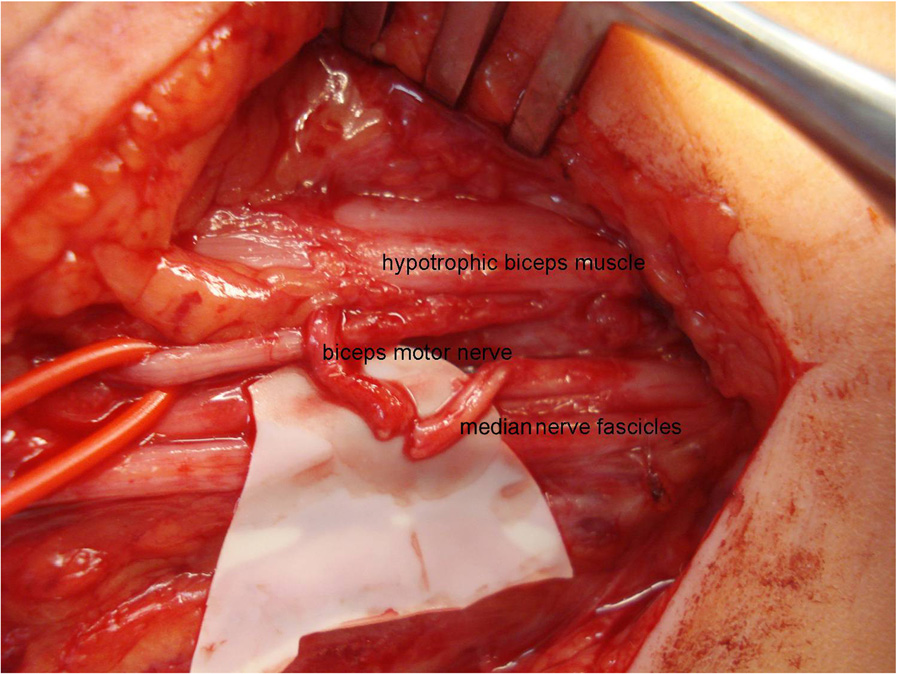
Patient 2 intraoperative (Oberlin transfer).

Boy patient 2 was born with typical AMC affecting all four limbs. No active elbow flexion was present at 15 months and we decided together with the parents to explore the *right* upper arm to verify the presence of the biceps muscle and to try to functionally reanimate the elbow flexors by a fascicular ulnar nerve transfer [3], targeting the motor branch of the hypotrophied biceps brachii muscle (Figure 3).

Exploration was performed when he was 21 months old using an anterior approach of the upper arm, showing a good muscle bulk corresponding to the biceps brachii muscle and the presence of a rather thin musculocutaneous nerve, silent on direct electrical stimulation. We identified the motor branch directed to the biceps brachii muscle and performed a typical nerve transfer according to the technique described by Oberlin [3], using motor fascicles out of the median nerve to target the biceps motor branch (Figure 3a and Figure 3b). Six months postoperatively, the boy started to show active elbow flexion (Additional file 1: Video ten months postoperatively), an active movement pattern never shown before. The recovery is actually continuing 20 months after the nerve transfer.

## Discussion

These two cases illustrate that upper limb AMC may be associated with brachial plexus root hypoplasia, like seen in traumatic partial root avulsions. All five roots in patient 1 were thin and did only partially and weakly respond to direct electrical stimulation. This pattern has been observed in children suffering from severe upper obstetric brachial plexus palsy after a breech delivery with proven partial or total root avulsions. True hypoplastic malformations or congenital abnormalities of the brachial plexus are not described in the literature; but are reported by surgeons with longlasting experience: Gilbert described 3 cases of brachial plexus malformation out of 1000 operated children [4].

**Figure 4:**
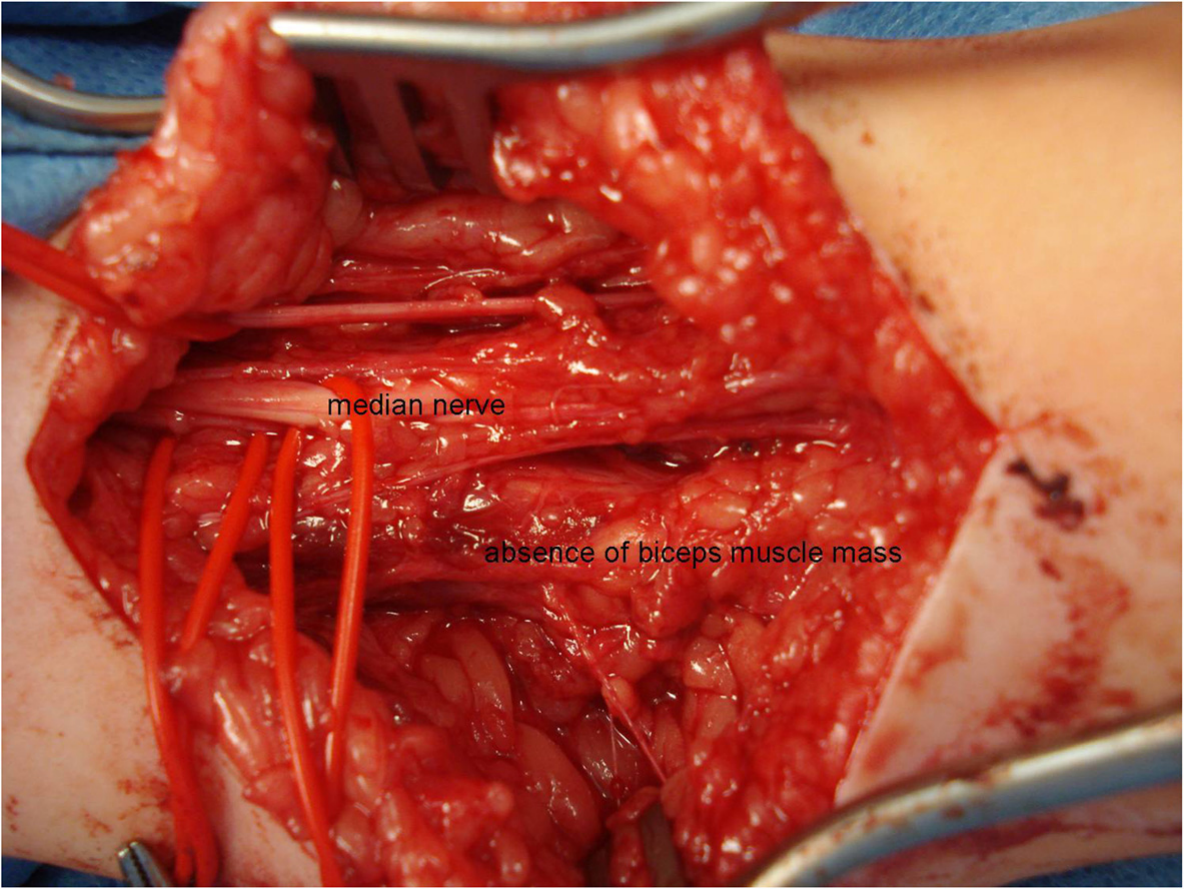
Other child: no functional biceps mass present.

In our clinic, after these first two cases with specific upper limb involvement, presence of a good muscle mass with absent or poor motor innervation has since been verified in three other children (Figure 4). Only in the here presented patient 2, the muscle mass was sufficient to expect a functional reinnervation through a fascicular motor nerve transfer. So far, the postoperative evolution shows an increase in active elbow flexion, hopefully ending up with a strength M3-M4 rendering a secondary muscle transfer of the latissimus dorsi or pectoralis muscle unnecessary.

Selective motor nerve transfers thus might be helpful in these children, if enough muscle mass is present at the shoulder or arm level and if dispensable motor nerve donors are available, without compromising the existing and sometimes weaker than normal motor functions. Obviously, the challenge in patient 2 was to avoid downgrading the overall good hand function, which did not appear on global hand function assessment postoperatively.

## Conclusion

Nerve transfers prior to muscle transfers could change the prognosis and functional outcome in selected AMC children, as morphologically developed target muscles even with poor motor innervation could be salvaged and functionally upgraded. Muscle transfer options still remain possible, even at an early moment.

## Consent

Written informed consent was obtained from the patients’ parents for publication of both case reports and any accompanying images and videos. A copy of the written consent for each case report is available for review by the editorial office.

## Competing interest

The author declares he has no competing interests.

## Supplementary Material

Additional file 1 **Video.** Patient 2 right upper limb function after surgery. Click here for file
